# Two Novel Relative Double-Stranded RNA Mycoviruses Infecting *Fusarium poae* Strain SX63

**DOI:** 10.3390/ijms17050641

**Published:** 2016-04-30

**Authors:** Luan Wang, Jingze Zhang, Hailong Zhang, Dewen Qiu, Lihua Guo

**Affiliations:** State Key Laboratory for Biology of Plant Disease and Insect Pests, Institute of Plant Protection, Chinese Academy of Agricultural Sciences, Beijing 100193, China; wluan@iccas.ac.cn (L.W.); jingzezhang0820@gmail.com (J.Z.); zhanghailong.shenyang@gmail.com (H.Z.); qiudewen@caas.cn (D.Q.)

**Keywords:** novel mycovirus, *Fusarium poae*, dsRNA virus, *Fusagraviridae*, FpV2, FpV3

## Abstract

Two novel double-stranded RNA (dsRNA) mycoviruses, termed Fusarium poae dsRNA virus 2 (FpV2) and Fusarium poae dsRNA virus 3 (FpV3), were isolated from the plant pathogenic fungus, *Fusarium poae* strain SX63, and molecularly characterized. FpV2 and FpV3, with respective genome sequences of 9518 and 9419 base pairs (bps), are both predicted to contain two discontinuous open reading frames (ORFs), ORF1 and ORF2. A hypothetical polypeptide (P1) and a RNA-dependent RNA polymerase (RdRp) are encoded by ORF1 and ORF2, respectively. Phytoreo_S7 domain (pfam07236) homologs were detected downstream of the RdRp domain (RdRp_4; pfam02123) of the ORF2-coded proteins of both FpV2 and FpV3. The same shifty heptamers (GGAAAAC) were both found immediately before the stop codon UAG of ORF1 in FpV2 and FpV3, which could mediate programmed –1 ribosomal frameshifting (–1 PRF). Phylogenetic analysis based on RdRp sequences clearly place FpV2 and FpV3 in a taxonomically unassigned dsRNA mycovirus group. Together, with a comparison of genome organization, a new taxonomic family termed *Fusagraviridae* is proposed to be created to include FpV2- and FpV3-related dsRNA mycoviruses, within which FpV2 and FpV3 would represent two distinct virus species.

## 1. Introduction

Mycoviruses, or fungal viruses, selectively infect fungi and are widespread in all major taxonomic fungal groups [1]. Most mycoviruses contain either double-stranded (ds) RNA or positive single-stranded (ss) RNA genomes, and one has an ssDNA genome, Sclerotinia sclerotiorum hypovirulence-associated DNA virus 1 (SsHADV-1) [2]. Negative ssRNA viruses were found recently by strong evidence from RNA sequencing analysis [3,4]. At present, mycoviruses are classified into 13 families. Six of these are composed of ssRNA genomes (*Hypoviridae*, *Narnaviridae*, *Alphaflexiviridae*, *Gammaflexiviridae*, *Endornaviridae*, and *Barnaviridae*), five consist of dsRNA genomes (*Reoviridae*, *Partitiviridae*, *Chrysoviridae*, *Totiviridae*, and *Megabirnaviridae*), and the other two families hold RNA reverse-transcribing genomes (*Metaviridae* and *Pseudoviridae*) [5]. In addition, several dsRNA species have been unassigned to any genus or family; for example, Botrytis cinerea RNA virus 1 (BcRV1), Fusarium graminearum dsRNA mycovirus-3 (FgV3), Fusarium virguliforme dsRNA mycovirus 1 and 2 (FvV1 and FvV2), Macrophomina phaseolina dsRNA virus 2 (MpRV2) and Sclerotinia sclerotiorum dsRNA mycovirus-L (SsNsV-L); these are large monopartite viruses representing an entirely different evolutionary lineage of dsRNA viruses [6,7,8,9].

In most cases, mycovirus infection causes little or inconspicuous symptoms in their host [1]; nevertheless, infection with some mycoviruses in the families *Megabirnaviridae*, *Hypoviridae*, *Partitiviridae*, *Narnaviridae*, and *Reoviridae*, or the unassigned negative-strand ssRNA and ssDNA mycoviruses, can cause clearly abnormal symptoms in the host, such as reducing mycelial growth, decreasing production of spores and/or sclerotia, suppressing biosynthesis of secondary metabolites, and attenuating aggressiveness or virulence [2,4,10,11,12]. The positive-strand ssRNA mycovirus Cryphonectria hypovirus 1 (CHV1) in the *Hypoviridae* family is a classic example and has been reported as a promising biocontrol agent for combating chestnut blight [13]. In addition, detailed research on the relationship of mycoviruses and their fungal hosts can provide a new insight into the molecular pathogenesis of plant-pathogenic fungi [12].

The hosts of dsRNA mycoviruses contain many *Fusarium* species. These filamentous fungi include important plant pathogens [14] that cause fusarium head blight (FHB), a serious disease that damages economically-important crops, such as maize, wheat, and barley. *Fusarium* spp. also produce mycotoxins that may have a negative impact on public health [15]. The frequency of occurrence of dsRNAs is high in *F. poae* but low in other *Fusarium* species. Among 55 *F. poae* isolates collected from wheat in different geographical regions in the world, all contained dsRNAs and encapsidated virus-like particles [16]. In addition, although the patterns of dsRNAs were different in each *F. poae* isolate, they were stable after repeated subculturing [16]. None of the *F. poae* isolates containing dsRNAs displayed any morphological changes, which demonstrates that the mycoviruses do not greatly harm the host. One of these mycoviruses, obtained from *F. poae* isolate A-11, was later identified as Fusarium poae virus 1 (FpV1), a member of the genus *Partitivirus*—which was the only report of the complete genome sequence of mycovirus from *F. poae* [17].

In this study, we present the sequence and molecular characteristics of two novel relative dsRNA mycoviruses, termed Fusarium poae dsRNA virus 2 (FpV2) and Fusarium poae dsRNA virus 3 (FpV3), both isolated from identical *Fusarium poae* strain SX63. We recommend that FpV2 and FpV3, together with the previously-reported SsNsV-L, BcRV1, FgV3, MpRV2, FvV1, and FvV2, are placed in a group, for which a new family *Fusagraviridae* is proposed.

## 2. Results and Discussion

### 2.1. Detection and Complete Genome Sequencing of dsRNA in Fusarium poae Strain SX630

**Figure 1:**
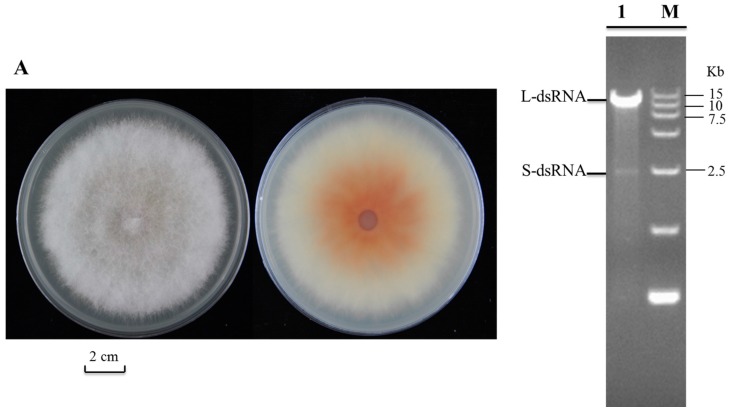
Identification of dsRNA isolated from *Fusarium poae*. (**A**) Colony morphology of strain SX63 after three days of culture on PDA at 25 °C in the dark; (**B**) Agarose gel electrophoresis of dsRNA isolated from *Fusarium poae* strain SX63. The nucleic acid was fractionated on 1.0% agarose gel and stained with ethidium bromide. Lane M, DNA marker (15-kb ladder, TaKaRa); lane 1, dsRNA sample with treatment of both RNase-free DNase I and S1 nuclease.

Strain SX63 (Figure 1A), which was originally isolated from the disease glumes of wheat infected with *Fusarium* spp., was identified as *F. poae* by PCR amplification of the translation elongation factor (EF-1) fragment [18] (data not shown). dsRNA was isolated from mycelial extracts of strain SX63 and its agarose gel electrophoresis showed the presence of two distinct dsRNA segments (L-dsRNA and S-dsRNA), which were *ca.* 9 and 2.5 kb, respectively (Figure 1B). Both segments were confirmed to be dsRNA in nature according to resistance to DNase I and S1 nuclease (Figure 1B). The L-dsRNA band was agarose gel-purified and then undergone cDNA synthesis, PCR amplification, cloning, and sequencing as described by Li *et al.* [19]. Computer-assisted sequence assembly revealed that the L-dsRNA was virtually a doublet component of two co-migrating dsRNA segments; therefore, the resulting L-dsRNA was assigned to L-1 and L-2 dsRNA. The full-length sequence of L-1 and L-2 dsRNA are 9518 and 9419 bp, respectively, lacking the poly (A) tail at their 3′-terminal, which were also confirmed by RT-PCR with specific primers. The mycoviral L-1 and L-2 dsRNA were tentatively assigned the name “Fusarium poae dsRNA virus 2 (FpV2)” and “Fusarium poae dsRNA virus 3 (FpV3)”, respectively. These names refer to Fusarium poae virus 1 (FpV1), a member of the genus *Partitivirus*, which was the only report of the complete genome sequence of mycovirus from *F. poae* [17]. The sequences of FpV2 and FpV3 were deposited in GenBank under accession numbers KU728180 and KU728181, respectively.

### 2.2. Both FpV2 and FpV3 Have a Double-Stranded RNA Genome

**Figure 2:**
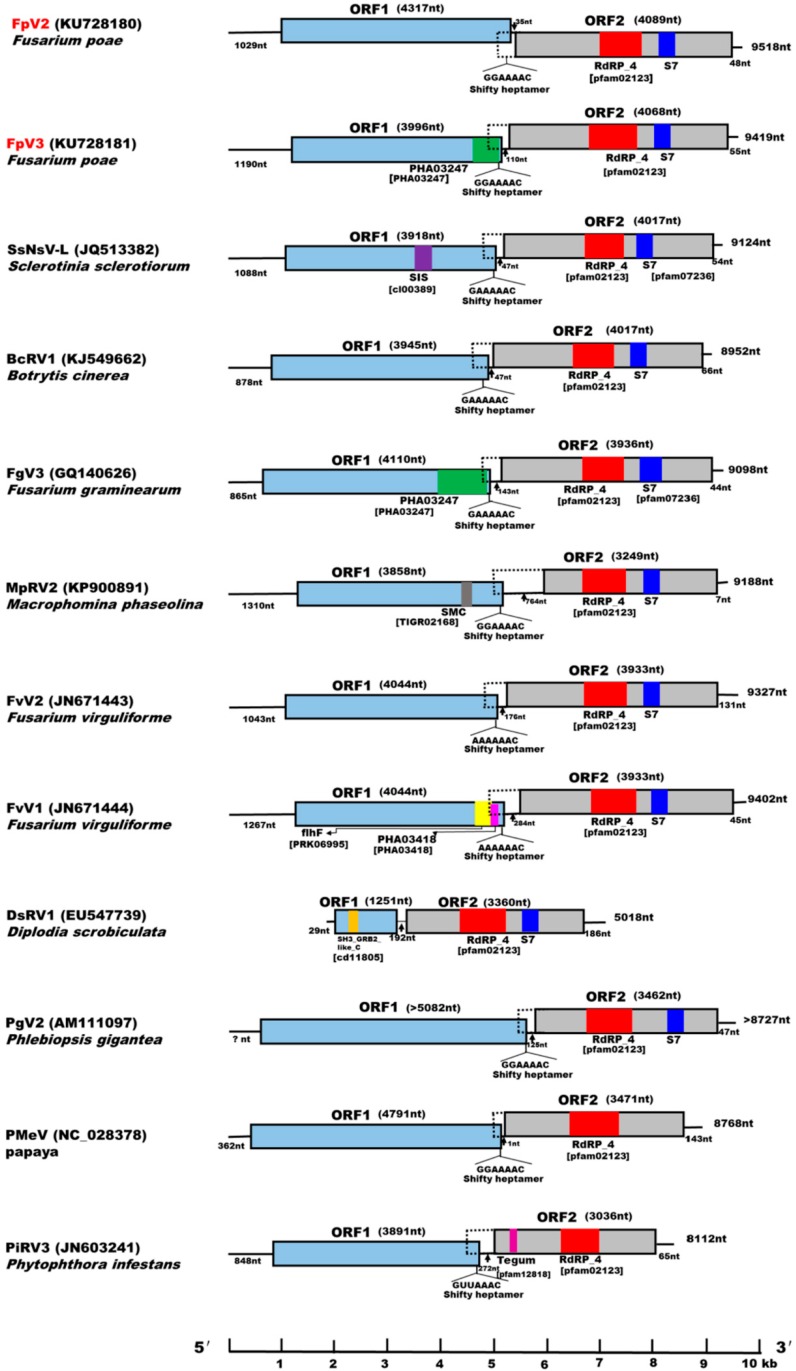
Genomic organization of FpV2, FpV3 and comparison with those of related RNA viruses. The genomes of FpV2 and FpV3 are 9518 and 9419 nt in length with two open reading frames (ORFs), respectively. The dotted line box represents a possible extension of ORF2 by frameshifting. The colored boxes and lines stand for ORFs and non-coding sequences, respectively. The boxes in ORF 2 indicate conserved domains containing RNA-dependent RNA polymerase superfamily 4 (RdRP_4, red) and (or) Phytoreo_S7 (S7, blue). The sizes of the 5′-UTR, 3′-UTR, inter-ORF regions, ORF1, and ORF2 are shown above or below the diagram. A scale bar (1-kb increment) is shown at the bottom. See legend to Figure 4 for abbreviations of the virus names.

The genome sequence analysis showed that both FpV2 and FpV3 contain two discontinuous, large, open reading frames (ORFs): ORF1 and ORF2 on their genomic plus strand, with long 5′-UTRs of 1029 and 1190 bp, and relatively short 3′-UTRs of 48 and 55 bp, respectively (Figure 2 and Table 1). The 5′-proximal ORF1 of FpV2 and FpV3 (nt 1030–5346 and nt 1191–5186, respectively) putatively encode proteins of 1437 and 1330 amino acids (aa) with predicted molecular masses of 160.6 and 147.8 kDa, respectively (Figure 2). A sequence search with BLASTP showed that both have a low percentage of sequence similarity (22%–49%) to the hypothetical protein (P1) of eight unclassified dsRNA viruses in the database: BcRV1 [6], FgV3 [7], FvV1 [8], FvV2 [8], MpRV2, SsNsV-L [9], Grapevine associated totivirus-2 (GaTV2) [20], and Phlebiopsis gigantea mycovirus dsRNA 2 (PgV2) [21] (Table 2). In addition, the sequence of P1 of FpV2 also shares some sequence similarity with the dsRNA virus, Papaya meleira virus (PmeV) [22] (Table 2). No putative conserved domains were found in the ORF1 protein of FpV2 using the Conserved Domain Databases (CDD) search program on the National Center for Biotechnology Information (NCBI) website. However, a search of the CDD showed that the ORF1 of FpV3 has a significant match (*E*-value = 6.05 × 10^−^^5^) with a partial consensus sequence of the provisional large tegument protein UL36 (PHA03247; PHA03247), which is conserved in the large tegument protein in the family *herpesviridae* (Figure 2). Nevertheless, the function of ORF1 protein is still unclear.

**Figure 3:**
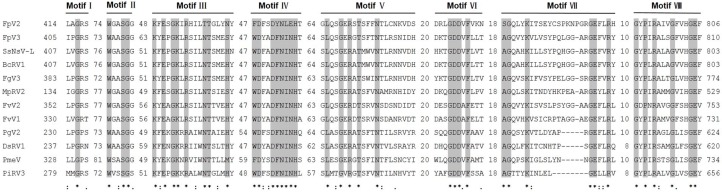
Multiple alignments of the amino acid sequences of the conserved motifs in the ORF2-coded RNA dependent RNA polymerase (RdRp) of FpV2, FpV3, and related viruses in the possible unassigned family. See legend to Figure 4 for abbreviations of the virus names. Motif I–VIII involves the eight conserved motifs characteristic of RdRps of RNA viruses. Identical residues are shaded. Asterisks, colons, and dots indicate identical amino acid residues (shaded gray), conserved amino acid residues, and semi-conserved amino acid residues, respectively. Numbers in gaps represent the number of amino acid residues apart from the motifs.

The 3′-proximal ORF2 of FpV2 and FpV3 (4089 and 4068 nt, respectively) potentially encode 1361 and 1354 aa proteins with predicted molecular masses of 152.6 and 149.4 kDa, respectively. These proteins have a low percentage of sequence similarity (28%–44%) to the putative RNA-dependent RNA polymerases (RdRps) of the same nine unclassified viruses mentioned above, as well as two other unclassified dsRNA viruses found in a BLASTP search: Diplodia scrobiculata RNA virus 1 (DsRV1) [23] and Phytophthora infestans RNA virus 3 (PiRV3) [24] (Figure 2 and Table 2). CDD searches and multiple protein alignment verified that the predicted ORF2 of both FpV2- and FpV3-coded proteins contain a conserved RdRp domain (RdRp_4; pfam02123) having eight conserved motifs (I–VIII) characteristic of the RdRps in dsRNA viruses [25] (Figure 3). The similarities in the RdRp regions suggest that FpV2 and FpV3 are dsRNA viruses. The predicted protein sequences of the two ORFs of FpV2 and FpV3 share 24.27% and 29.44% identity, respectively. Hence, FpV2 and FpV3 represent two distinct virus species (Table 1).

### 2.3. Phylogenetic Analysis Based on the RdRp and P1 Sequences

**Figure 4:**
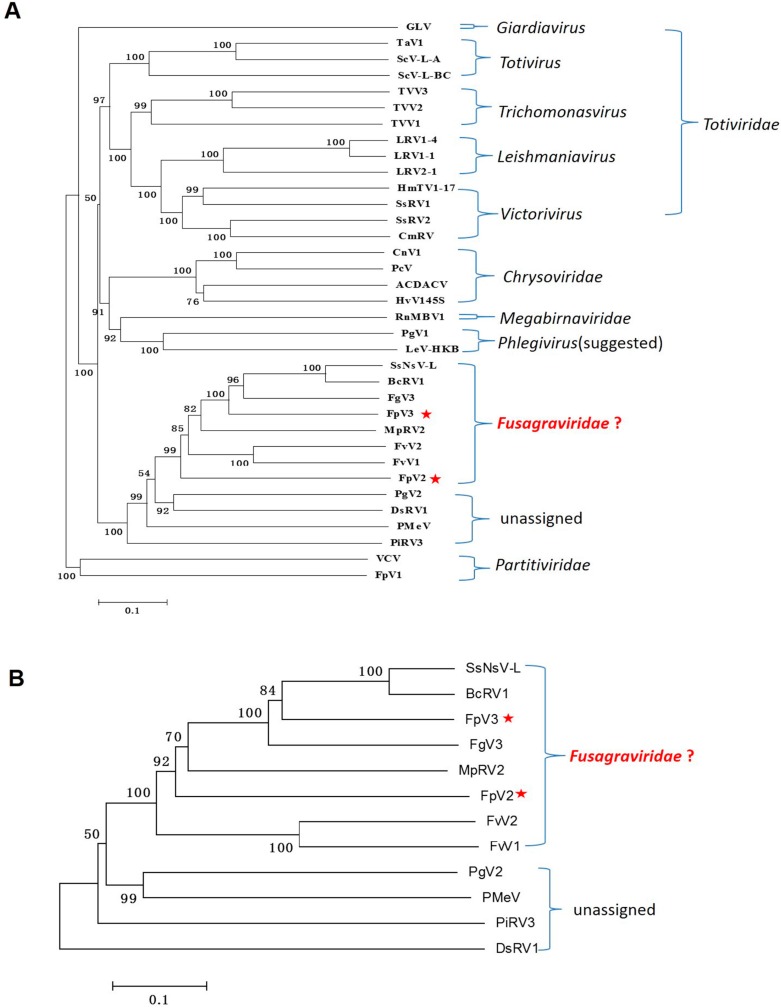
Phylogenetic analysis of FpV2, FpV3 (marked by stars), and related RNA viruses. The phylogenetic tree generated by NJ method (1000 bootstrap replicates) based on the amino acid sequences of the putative RdRp regions (**A**) and the polypeptide P1 (**B**) in MEGA 6.0. In (**A**), two partitivirus sequences (FpV1and VCV) were included as an outgroup, on which the tree is rooted. The scale bar is equivalent to a genetic distance of 0.1 amino acid substitutions per site. “?” means the proposed family name is waiting for approval of ICTV. The abbreviation of virus names and GenBank accession numbers are as follows: ACDACV, Amasya cherry disease-associated chrysovirus (CAG77602); BcRV1, Botrytis cinerea RNA virus 1 (AIW58875); CmRV, Coniothyrium minitans RNA virus (AAO14999); CnV1, Cryphonectria nitschkei chrysovirus 1 (ACT79255); DsRV1, Diplodia scrobiculata RNA virus 1 (ACD91658); FgV3, Fusarium graminearum dsRNA mycovirus 3 (ACY56323); FpV1, Fusarium poae virus 1 (AAC98734.1); FvV1, Fusarium virguliforme dsRNA mycovirus 1 (AEZ54148); FvV2, Fusarium virguliforme dsRNA mycovirus 2 (AEZ54146); GLV, Giardia lamblia virus (NP_620070); HmTV1-17, Helicobasidium mompa dsRNA virus 17 (BAC81754); HvV145S, Helminthosporium victoriae 145S virus (YP_052858); LeV-HKB, Lentinula edodes mycovirus HKB (AEB96150); LRV1-1, Leishmania RNA virus 1-1 (NP_041191); LRV1-4, Leishmania RNA virus 1-4 (NP_619653); LRV2-1, leishmania RNA virus 2-1 (AAB50031); MpRV2, Macrophomina phaseolina double-stranded RNA virus 2 (ALD89097); PcV, Penicillium chrysogenum virus (AAM95601); PgV1, Phlebiopsis gigantea mycovirus dsRNA 1 (CAJ34333); PgV2, Phlebiopsis gigantea mycovirus dsRNA 2 (CAJ34335); PiRV3, Phytophthora infestans RNA virus 3 (AEX87902); PmeV, Papaya meleira virus (YP_009179230); RnMBV1, Rosellinia necatrix megabirnavirus 1 (BAI48016); ScVL-A, Saccharomyces cerevisiae virus L-A (AAA50508); ScVL-BC, Saccharomyces cerevisiae virus L-BC (NP_042581); SsNsV-L, Sclerotinia sclerotiorum nonsegmented virus L (YP_006331065); SsRV1, Sphaeropsis sapinea RNA virus 1 (AAD11601); SsRV2, Sphaeropsis sapineaRNAvirus 2 (AAD11603); TaV1, Tuber aestivum virus 1 (ADQ54106.1); TvV1, Trichomonas vaginalis virus 1 (AAA62868); TvV2, Trichomonas vaginalis virus 2 (AAF29445); TvV3, Trichomonas vaginalis virus 3 (AKE98372); VCV, Vicia cryptic virus (ABN71234.1).

A phylogenetic tree was constructed using the neighbor-joining (NJ) method based on the RdRp sequences of FpV2, FpV3, and 33 selected RNA viruses (Figure 4A). Together with 14 members of the family *Totiviridae*, four members of the family *Chrysoviridae*, two members of the genus *Phlegivirus* (suggested) [26], the unassigned dsRNA viruses recognized through BLAST searching, and an additional three dsRNA viruses from the families *Megabirnaviridae* and *Partitiviridae* were subjected to phylogenetic analysis. It was showed that FpV2 and FpV3 form a clear, well-supported taxonomic cluster together with SsNsV-L, BcRV1, FgV3, MpRV2, FvV2, FvV1, PgV2, DsRV1, PMeV, and PiRV3 in the unclassified dsRNA viruses. This clade is closely relevant with the clades for *Phlegivirus*, *Megabirnaviridae*, *Chrysoviridae*, and *Totiviridae*. However, it is distantly related to the clade for *Partitiviridae*. Results also showed that within the unassigned clade, FpV2 is placed between the FvV1 and PgV2 branches, and FpV3 is placed between the FgV3 and MpRV2 branches. The putative RdRp encoded by FpV2 shares the highest sequence identity with that of MpRV2 (36%) and PgV2 (36%), respectively, while RdRp encoded by FpV3 shares the highest sequence identity with that of FgV3 (44%), SsNsV-L (44%), and GaTV2 (44%), respectively (Table 2).

Furthermore, phylogenetic analysis based on the viral P1 protein clearly showed that the unassigned dsRNA virus clade includes more than two groups, with one of them grouping FpV2, FpV3, SsNsV-L, BcRV1, FgV3, MpRV2, FvV1, and FvV2 (Figure 4B), supporting the phylogenetic tree results based on the RdRp sequences (Figure 4A). The putative polypeptide P1 encoded by FpV2 shares the highest sequence identity with that of MpRV2 (34%), while P1 encoded by FpV3 shares the highest sequence identity with that of SsNsV-L (49%) (Table 2). These comparisons suggest that FpV2 and FpV3 belong to an unassigned dsRNA virus group, which may represent a new mycovirus family.

### 2.4. Identification of “Phytoreo_S7 Domain” in FpV2, FpV3, and Related Unassigned dsRNA Viruses

**Figure 5:**
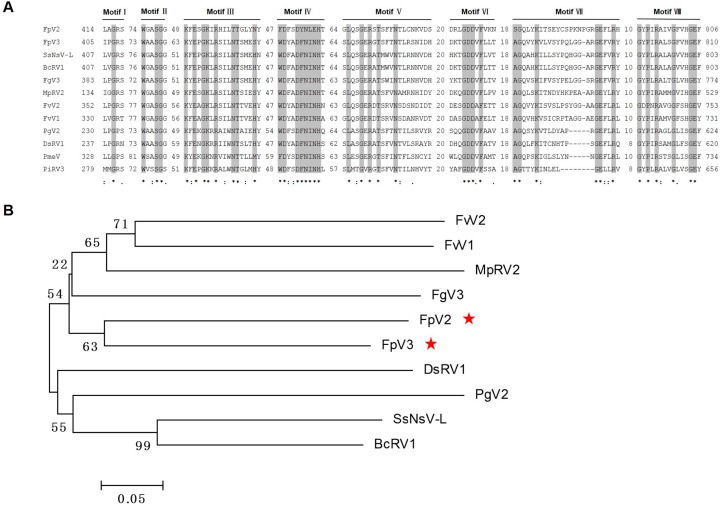
(**A**) Multiple alignments of the homologs of Phytoreo_S7 domain from FpV2, FpV3, and related viruses. Asterisks, colons, and dots represent identical amino acid residues (shaded gray), conserved amino acid residues, and semi-conserved amino acid residues, respectively; (**B**) Phylogenetic tree of the Phytoreo_S7 domain homologs from FpV2, FpV3 (marked by stars) and eight related RNA viruses. The phylogenetic tree was generated by NJ method (1000 bootstrap replicates) in MEGA 6.0. See legend to Figure 4 for abbreviations of the virus names.

The Phytoreo_S7 domain is characteristic of a protein family composed of several phytoreovirus S7 proteins that are viral core proteins with the activities of binding nucleic acid. The Phytoreo_S7 domain (pfam07236) were detected by CDD searches only in SsNsV-L [9] and FgV3 [7], but not in FpV2, FpV3, and related unassigned dsRNA viruses (Figure 2). Furthermore, multiple protein alignment confirmed that a region of ORF2 (aa 927 to 1029) of FpV2 and ORF2 (aa 908 to 1010) of FpV3 downstream of the RdRp domain have significant sequences similar to a consensus sequence of the Phytoreo_S7 domain (pfam07236) (Figure 2 and Figure 5A). Homologous S7 domain polypeptides were also identified in BcRV1 [6], MpRV2, FvV1, FvV2, DsRV1, and PgV2, but not in PMeV and PiRV3, by multiple protein alignment of ORF 2 amino acids (Figure 2 and Figure 5A). The phylogenetic tree based on the Phyto_S7 domain of FpV2 and FpV3 and related dsRNA viruses was not consistent with the tree based on the RdRp domain (Figure 4A and Figure 5B). Results showed that the Phytoreo_S7 domain sequences of FpV2 and FpV3 are closely related (Figure 5B). In addition, Liu *et al.* [9] revealed that S7 domain homologs are widely distributed in members of *Chrysoviridae* and *Endornaviridae* through multiple horizontal gene transfer (HGT) events among diverse virus groups. The function of this domain in these non-phytoreoviruses is not known. Interestingly, the S7 domain is located downstream of the RdRp domain of ten members of the closely-related 12 unclassified viruses, including FpV2, FpV3, SsNsV-L, FgV3, BcRV1, MpRV2, FvV1, FvV2, DsRV1, and PgV2. However, the S7 domain is located upstream of the RdRp domain in *Endornaviruses* and *Chrysoviruses* [9]. A similar arrangement of these S7 and RdRp domains suggest that these ten unclassified dsRNA mycoviruses may have a common ancestor containing the S7 domain.

### 2.5. Potential Programmed –1 Ribosomal Frameshifting (–1 PRF) in FpV2, FpV3, and Related Unassigned dsRNA Viruses

Programmed –1 ribosomal frameshifting is a common translational recoding mechanism in RNA viruses as same as in other organisms [27]. When –1 PRF occurs, translating ribosomes shift one residue backward at a slippery sequence and then continue translation in the new –1 reading frame, generating an extension product [28]. Efficient eukaryotic –1 PRF requires cis-acting frameshift signals typically composed of a heptameric slippery sequence, X XXY YYZ (X is any nucleotide, Y is either A or U, and Z is not G) and a Recoding Stimulatory Element (RSE) positioned just downstream from the slippery site. The RSE is often an H-type pseudoknot, a stable imperfect hairpin, or a large bulged hairpin [29,30]. Candidate shifty heptamers (GGAAAAC, nt 5337 to 5343 of FpV2 and nt 5177 to 5183 of FpV3) were both found immediately before the stop codon UAG of ORF1 in FpV2 and FpV3. This could allow tRNAs in the A-site and P-site of the translating ribosome to un-pair from 0 frame codons and re-pair with at least two of three residues in –1 frame codons during the frameshift event [31] (Figure 2). Except for DsRV1, all the dsRNA viruses in the unclassified group (SsNsV-L, BcRV1, FgV3, MpRV2, FvV2, FvV1, PgV2, PMeV, and PiRV3) have been identified to contain a putative shifty heptamer motif located immediately upstream of the stop codon of ORF1 (Figure 2). Candidate RSE structures have been identified just downstream of the slippery site in FpV2, FpV3, and PMeV using Mfold (version 2.3 energies, SUNY Albany Research IT Group, Albany, CA, USA) [32] (data not shown) and eight other relatives [6]. FpV2 and FpV3 have the same shifty heptamer (GGAAAAC) with three related viruses: MpRV2, PgV2, and PMeV. Remarkably, among these related unassigned viruses, PMeV is the only virus that infects plants. Furthermore, the first three nucleotides in the shifty heptamers for FpV2 and FpV3 and their relatives are variable (GGA in FpV2, FpV3, MpRV2, PgV2, and PMeV; GAA in BcRV1 and FgV3; AAA in FvV1 and FvV2; and GUU in PiRV3), however, the last four nucleotides (AAAC) are highly conserved. The first three nucleotides in the shifty heptamer have been reported to have greatly effect on the efficiency of the ribosomal frameshifting during the expression of the totivirus Saccharomyces cerevisiae virus L-A (ScVL-A) [33]. The sequence similarities of the shifty heptamer among these unclassified viruses suggest they may have a common ancestor utilizing –1 PRF. However, of the related unclassified viruses, only the shifty heptamer and RSE structure of BcRV1 have been proved to be able to mediate –1 PRF in the *Escherichia coli* expression system [6]. Thus, further studies are necessary to prove the –1 PRF translational recoding mechanism and to elucidate the functions of different shifty heptamers in FpV2, FpV3, and other relative viruses.

### 2.6. A Proposition to Create a New Family Designated Fusagraviridae

A phylogenetic tree based on the RdRp sequences grouped FpV2 and FpV3 with SsNsV-L, BcRV1, FgV3, MpRV2, FvV1, FvV2, PgV2, DsRV1, PMeV, and PiRV3 in a separate clade distinct from other known dsRNA mycovirus families, including *Megabirnaviridae*, *Chrysoviridae*, and *Totiviridae* (Figure 4A). Furthermore, of the unclassified dsRNA viruses, FpV2, FpV3, SsNsV-L, BcRV1, FgV3, MpRV2, FvV1, and FvV2 were clustered into a clade distinct from PgV2, PMeV, PiRV3, and DsRV1 based on the phylogenetic trees of the RdRp and P1 sequences, respectively (Figure 4A,B). We propose establishing a novel family designated as *Fusagraviridae* (an acronym from the *Fusarium graminearum*, host of FgV3, the first reported related virus) containing FpV2, FpV3, SsNsV-L, BcRV1, FgV3, MpRV2, FvV1, and FvV2 viruses. The proposed family can be readily distinguished from other known mycovirus families on account of the size of their monopartite genomes (8112~9518 bp), the genomic structure with putative –1 PRF translational recoding mechanism, a long 5′-UTR (865–1310 bp) and a relatively short 3′-UTR (7–131 bp), and the arrangement of S7 and RdRp domains (Figure 2). Of the two proteins encoded by members of the family, excluding –1 PRF expression strategy, only the RdRp sequence displays low levels (around 20%–30%) of identity (data not shown) with those of members in the families *Megabirnaviridae*, *Chrysoviridae*, and *Totiviridae*, while the P1 proteins do not display any significant sequence similarities with other mycovirus proteins. To date, only FgV3 has been reported possibly not to form true virions [7]. Therefore, it is unclear whether the members of the proposed family form virions or not and needs further investigation.

In addition, of the unclassified dsRNA viruses, DsRV1, PMeV, PiRV3, and PgV2 differ from the members of the proposed family in the following aspects: DsRV1 has a relative short genome of 5018 bp in length and a short 5′-UTR of 29 bp, which has no putative shifty heptamer motif located immediately upstream from the stop codon of ORF1. PiRV3 and PMeV have not been identified as having a homologous S7 domain. Moreover, among the unassigned dsRNA viruses, PMeV, which has a short 5’-UTR of 362 bp, is the only virus that infects plants. PgV2 has an incomplete genome sequence, a partially-characterized dsRNA virus from *Phlebiopsis* *gigantea*. When completely characterized, these viruses may stand for additional species in the new genus or possibly new genera in the family.

Although such commonalities and their close phylogenetic relationship among FpV2, FpV3, SsNsV-L, BcRV1, FgV3, MpRV2, FvV1, and FvV2 viruses lead us to propose a new family, they have markedly different features. First, both sequence identities of P1 and RdRp are low in the range of 20.99%–47.29% (P1) and 26.46%–43.27% (RdRp), respectively, among the members of the family *Fusagraviridae*, except for those between BcRV1 and SsNsV-L (76.98% for P1 and 71.45% for RdRp) (Supplementary Tables S1 and S2); Second, different putative conserved domains were detected by CDD searches of the ORF1 protein of the members of the proposed family *Fusagraviridae*, including PHA03247 in FpV3 (*E*-value = 6.05 × 10^–5^) and FgV3 (*E*-value = 4.26 × 10^−3^), SMC (Structural Maintenance of Chromosome) (TIGR02168, *E*-value = 6.51 × 10^–3^) in MpRV2, which proteins have the ability of binding DNA and acting in organizing and segregating chromosomes for partition; SIS (Sugar ISomerase) (cl00389, *E*-value = 1.80 × 10^−3^) in SsNsV-L, which exist in many phosphosugar isomerases and phosphosugar binding proteins, and in proteins that regulate the expression of genes referred to synthesis of phosphosugars; flhF (flagellar biosynthesis regulator FlhF) (PRK06995, *E*-value = 1.54 × 10^−3^) and PHA03418 (hypothetical E4 protein) (*E*-value = 6.17 × 10^−4^) in FvV1 (Figure 2); Third, FpV2 and FpV3 have the largest genome with 9518 bp and 9419 bp in length, respectively, as compared with the other viruses of the family. In addition, MpRV2 has the longest 5′-UTR (1310 bp) and the shortest 3′-UTR (7 bp), whereas FgV3 has the shortest 5′-UTR (865 bp) and FvV2 has the longest 3′-UTR (131 bp) in family *Fusagraviridae* (Figure 2 and Table 2). These differences suggest that all of the viruses FpV2, FpV3, SsNsV-L, BcRV1, FgV3, MpRV2, FvV1, and FvV2 can be identified as different species and there exist a wide diversity of viral replication and function on virus encoding-protein among the members of the family *Fusagraviridae*.

## 3. Materials and Methods

### 3.1. Fungal Strain and Culture Conditions

*Fusarium poae* strain SX63 was isolated from the disease glumes of wheat infected with *Fusarium* spp. collected in the Shanxi province of China. The *F. poae* strain SX63 was routinely cultured on potato dextrose agar (PDA) at 25 °C in the dark. Mycelial plugs were stored in 25% glycerol at −80 °C. Small mycelial agar plugs were grown on top of cellophane membranes overlaid on PDA plates for three days at 25 °C in the dark. The mycelium was subsequently harvested for dsRNA extraction.

### 3.2. dsRNA Extraction and Purification

The harvested mycelial mass was ground in liquid nitrogen to a fine powder; the dsRNA was then extracted from the fine powder, following the CF-11 cellulose chromatography method previously described (Sigma-Aldrich, Dorset, UK) [34]. Total nucleic acid was purified by digestion with DNase I and S1 nuclease (TakaRa Bio Inc., Dalian, China) according to the manufacturer’s instructions to remove traces of DNA and ssRNA. The final dsRNA products were electrophoresed on 1.0% agarose gel and stained with ethidium bromide.

### 3.3. cDNA Synthesis, Molecular Cloning, and Sequencing

cDNA synthesis and molecular cloning of dsRNA were performed as previously described [35,36]. The RNA template was mixed with tagged random primers-dN6 (5’-GACGTCCAGATCGCGAATTCNNNNNN-3’), denatured at 95 °C for 10 min, and then chilled on ice for 5 min. The reverse transcriptase PCR (RT-PCR) procedure was carried out by using M-MLV reverse transcriptase (Promega, Madison, WI, USA) according to the manufacturer’s protocols. The resulting cDNAs were amplified by a specific primer (5′-GACGTCCAGATCGCGAATTC-3′) and PrimerSTAR^®^ HS DNA Polymerase (TaKaRa, Dalian, China) on a Thermal Cycler (Bio-Rad, Hercules, CA, USA) according to the manufacturer’s instructions. The products were fractionated on 1.0% agarose gel and agarose gel-purified with a gel extraction kit (Sigma, St. Louis, MO, USA). The resulting products were cloned into the PMD18-T vector (TaKaRa, Dalian, China) and transformed into Trans 5α Chemically Competent Cells (TransGen, Beijing, China). Positive clones were selected and sequenced by the Sanger method at the Beijing Genomics Institute (BGI, Shenzhen, China).

dsRNA-specific primers were designed for RT-PCR amplifications based on the obtained sequences in order to identify the gap sequences between different clones. RT-PCR amplifications were performed as previously described [35,36]. In brief, purified dsRNA and tagged random primers-dN6 were denatured at 95 °C for 10 min and then chilled on ice for 5 min. Reverse transcription was subsequently carried out by using Transcript™ II One-Step gDNA Removal and cDNA Synthesis SuperMix (TransGen, Beijing, China) at 50 °C for 2 h. The enzyme was then deactivated at 85 °C for 10 min. The resulting cDNA and specific primers were used 2× TransTaqs^®^ High Fidelity (HiFi) PCR SuperMix (TransGen, Beijing, China) for specific PCR amplification. PCR products were purified, cloned, and sequenced as previously described.

The 5′- and 3′-terminal sequences of dsRNA were determined to obtain the complete nucleotide sequences by a 3′ RNA Ligase-Mediated Rapid Amplification of cDNA Ends (RLM-RACE) protocol, as described by Xie *et al.* [37] and Chiba *et al.* [11]. All amplified cDNAs were cloned and sequenced as previously described.

The whole nucleotide sequences of dsRNA were completed and identified by overlapping of cDNA clones, and each base was ascertained by sequencing at least four independent clones.

### 3.4. Nucleotide Sequence Analysis

The resulting nucleotide sequences were assembled and analyzed using DNAMAN Software (Lynnon Biosoft, San Ramon, CA, USA). The translations of ORFs were also conducted using DNAMAN software, and potential ORFs were found by using the NCBI ORF Finder tool [38]. Protein domain searches were performed using NCBI’s CDD [39]. Searches for homologies were conducted using the online NCBI Blast program [40]. All of the genome and protein sequences of the dsRNA viruses involved in this study were downloaded from viral genome databases at the NCBI website.

### 3.5. Phylogenetic Analysis

Phylogenetic trees were estimated using the NJ method on the aligned amino acid sequences. The deduced amino acid sequences of the polyproteins or conserved domains were aligned using DNAMAN 6.0 Software with default parameters [41]. The NJ trees were constructed using MEGA 6.0 software [42] with a bootstrapping analysis of 1000 replicates.

### 3.6. GenBank Accession Number

The complete genomic sequences of dsRNA1 and dsRNA2 isolated from the strain SX63 have been deposited in the GenBank database with accession Nos. KU728180 and KU728181, respectively.

## 4. Conclusions

At present, mycoviruses are classified into 13 families, including *Hypoviridae*, *Narnaviridae*, *Alphaflexiviridae*, *Gammaflexiviridae*, *Endornaviridae*, *Barnaviridae*, *Reoviridae*, *Partitiviridae*, *Chrysoviridae*, *Totiviridae*, *Megabirnaviridae*, *Metaviridae*, and *Pseudoviridae* [5]. In addition, several dsRNA species have been unassigned to any genus or family; for example, BcRV1, FgV3, FvV1, FvV2, MpRV2, and SsNsV-L [6,7,8,9]. In this study, we characterized the molecular features of two novel dsRNA mycoviruses, Fusarium poae dsRNA virus 2 (FpV2) and Fusarium poae dsRNA virus 3 (FpV3), which were isolated from *F. poae* strain SX63. A comprehensive comparison of genome organization and a powerful phylogenetic analysis led us to recommend the creation of a novel family designated *Fusagraviridae* to include above mentioned FpV2- and FpV3-related dsRNA mycoviruses (BcRV1, FgV3, FvV1, FvV2, MpRV2, and SsNsV-L). The identification of FpV2 and FpV3 inevitably supports the suggestion of the assignation of a new virus family, in which FpV2 and FpV3 represent two distinct virus species.

## Figures and Tables

**Table 1:** Nucleotide and amino acid identities between FpV2 and FpV3.

Virus	Full Sequence		5′-UTR		3′-UTR		ORF1 (P1)		ORF2 (RdRP)	
	Length (bp)	nt %	Length (bp)	nt %	Length (bp)	nt %	nt %	aa %	nt %	aa %
FpV2	9518	42.97	1029	36.12	48	44.07	39.91	24.27	44.05	29.44
FpV3	9419		1190		55					

Abbreviations: ORF, open reading frame; UTR, untranslated region.

**Table 2:** BLASTP search results of ORF1-coded polypeptide P1 and ORF2-coded RNA-dependent RNA polymerase (RdRp) of FpV2 and FpV3.

Search Target And Virus Name (GenBank Accession No.) ^a^	Size (aa)	% Identity	Overlap (Positions)	Bit Score	*E-*Value
		FpV2 (FpV3)	FpV2 (FpV3)	FpV2 (FpV3)	FpV2 (FpV3)
P1 search					
MpRV2 (ALD89096)	1285	34 (38)	322/960 (340/885)	482 (558)	1 × 10^−142^ (9 × 10^–^^172^)
SsNsV-L (YP_006331064)	1305	33 (49)	353/1057 (619/1276)	424 (1125)	1 × 10^–^^121^ (0.0)
FgV3 (YP_003288788)	1369	32 (44)	328/1037 (538/1217)	423 (944)	8 × 10^−121^ (0.0)
GaTV2 (ADO60932)	1313	33 (47)	353/1076 (635/1351)	397 (1123)	2 × 10^−112^ (0.0)
BcRV1 (YP_009115497)	1314	32 (47)	347/1075 (644/1363)	395 (1118)	1 × 10^−111^ (0.0)
FvV2 (AEZ54145)	1347	27 (28)	295/1083 (257/910)	337 (329)	6 × 10^−92^ (1 × 10^−89^)
FvV1 (AEZ54147)	1311	26 (29)	281/1066 (286/983)	335 (357)	1 × 10^−91^ (5 × 10^−99^)
PgV2 (CAJ34334) ^b^	>1696	22 (28)	195/886 (117/424)	123 (124)	9 × 10^−25^ (4 × 10^−25^)
PmeV (YP_009179229)	1596	25 (ND)	93/365 (ND)	91.7 (ND)	5 × 10^−15^ (ND)
RdRp search					
MpRV2 (ALD89097)	1082	36 (37)	386/1076 (410/1116)	625 (664)	0.0 (0.0)
FgV3 (YP_003288789)	1311	32 (44)	421/1324 (605/1366)	568 (1041)	1 × 10^−174^ (0.0)
FvV1 (AEZ54148)	1289	30 (31)	401/1328 (408/1305)	556 (561)	2 × 10^−170^ (3 × 10^−172^)
BcRV1 (YP_009115498)	1338	31 (43)	427/1356 (594/1372)	556 (993)	5 × 10^−170^ (0.0)
SsNsV-L (CEZ26308)	1338	31 (44)	421/1338 (601/1370)	556 (1056)	7 × 10^−170^ (0.0)
FvV2 (AEZ54146)	1310	30 (31)	406/1346 (662/1398)	535 (558)	1 × 10^−162^ (6 × 10^−171^)
DsRV1 (YP_003359178)	1110	28 (32)	305/1094 (322/1005)	365 (436)	8 × 10^−103^ (5 × 10^−128^)
PgV2 (CAJ34335)	1153	36 (38)	226/632 (234/623)	331 (380)	5 × 10^−91^ (2 × 10^−107^)
PiRV3 (AEX87902)	1011	29 (31)	190/645 (188/614)	243 (241)	1 × 10^−62^ (8 × 10^−62^)
PmeV (YP_009179230)	1156	31 (33)	166/535 (197/604)	241 (280)	2 × 10^−61^ (3 × 10^−74^)
GaTV2 (ADO60933) ^b^	>613	31(44)	196/632 (274/626)	230 (456)	3 × 10^−60^ (6 × 10^−141^)

^a^ See legend to Figure 4 for abbreviations of the virus names; ^b^ The P1 of PgV2 and RdRp sequences of GaTV2 are incomplete in the NCBI; (FpV3), the BLASTP search results of ORF1-coded polypeptide P1 and ORF2-coded RdRp of FpV3; ND, not detected.

## Supplementary Materials

Supplementary materials can be found at http://www.mdpi.com/1422-0067/17/5/641/s1.
