# Supplementary material for: Two Novel Relative Double-Stranded RNA Mycoviruses Infecting Fusarium poae Strain SX63
Source: Int J Mol Sci. 2016 Apr 30;17(5):641. doi: 10.3390/ijms17050641 (PMC4881467; doi:10.3390/ijms17050641)
Supplement: Supplementary file 1 [file ijms-17-00641-s001.pdf]

# Two Novel Relative Double-Stranded RNA Mycoviruses Infecting *Fusarium poae* Strain SX63

**Luan Wang, Jingze Zhang, Hailong Zhang, Dewen Qiu and Lihua Guo ***

**Table S1.** Amino acid sequence identity (%) of the polypeptide P1 encoded by the members in the proposed family *Fusagraviridae*.

| | FpV2 | FpV3 | SsNsV-L | BcRV1 | FgV3 | FvV1 | FvV2 | MpRV2 |
|---|---|---|---|---|---|---|---|---|
| **FpV2** | 100 | 24.27 | 23.92 | 24.04 | 24.69 | 21.02 | 21.33 | 24.84 |
| **FpV3** | | 100 | 46.36 | 47.29 | 41.08 | 22.95 | 20.99 | 27.87 |
| **SsNsV-L** | | | 100 | 76.98 | 42.96 | 24.13 | 21.88 | 27.04 |
| **BcRV1** | | | | 100 | 42.58 | 24.40 | 22.53 | 26.65 |
| **FgV3** | | | | | 100 | 22.56 | 22.69 | 27.45 |
| **FvV1** | | | | | | 100 | 46.13 | 24.68 |
| **FvV2** | | | | | | | 100 | 24.08 |
| **MpRV2** | | | | | | | | 100 |

See legend to Figure 4 for abbreviations of the virus names.

**Table S2.** Amino acid sequence identity (%) of the RNA-dependent RNA polymerase (RdRp) encoded by the members in the proposed family *Fusagraviridae*.

| | FpV2 | FpV3 | SsNsV-L | BcRV1 | FgV3 | FvV1 | FvV2 | MpRV2 |
|---|---|---|---|---|---|---|---|---|
| **FpV2** | 100 | 29.44 | 27.79 | 28.38 | 28.31 | 26.67 | 27.12 | 27.04 |
| **FpV3** | | 100 | 41.98 | 42.23 | 43.00 | 28.31 | 29.21 | 28.51 |
| **SsNsV-L** | | | 100 | 71.45 | 43.20 | 27.97 | 26.46 | 29.02 |
| **BcRV1** | | | | 100 | 43.27 | 27.84 | 28.26 | 29.39 |
| **FgV3** | | | | | 100 | 28.03 | 26.86 | 28.78 |
| **FvV1** | | | | | | 100 | 42.38 | 27.85 |
| **FvV2** | | | | | | | 100 | 27.77 |
| **MpRV2** | | | | | | | | 100 |

See legend to Figure 4 for abbreviations of the virus names.
